# “Everything’s so Role-Specific”: VA Employee Perspectives’ on Electronic Health Record (EHR) Transition Implications for Roles and Responsibilities

**DOI:** 10.1007/s11606-023-08282-5

**Published:** 2023-10-05

**Authors:** Ellen A. Ahlness, Jay Orlander, Julian Brunner, Sarah L. Cutrona, Bo Kim, Brianne K. Molloy-Paolillo, Seppo T. Rinne, Justin Rucci, George Sayre, Ekaterina Anderson

**Affiliations:** 1 Center of Innovation for Veteran-Centered and Value-Driven Care, Seattle VA Medical Center, Seattle, WA USA; 2 Medical Service, VA Boston Healthcare System, Boston, MA USA; 3 Section of General Internal Medicine, Boston University Chobanian and Avedisian School of Medicine, Boston, MA USA; 4 Center for the Study of Healthcare Innovation, Implementation & Policy, VA Greater Los Angeles Health Care, Los Angeles, CA USA; 5 Center for Healthcare Organization and Implementation Research, VA Bedford Healthcare System, Bedford, MA USA; 6 Division of Health Informatics & Implementation Science, Department of Population and Quantitative Health Sciences, University of Massachusetts Chan Medical School, Worcester, MA USA; 7 Center for Healthcare Organization and Implementation Research, VA Boston Health Care System, Boston, MA USA; 8 Department of Psychiatry, Harvard Medical School, Boston, MA USA; 9 Division of Pulmonary and Critical Care Medicine, Department of Medicine, Boston University Chobanian and Avedisian School of Medicine, Boston, MA USA; 10 University of Washington School of Public Health, Seattle, WA USA

**Keywords:** EHR, EHR transitions, interface, care roles, role scope, United States Department of Veterans Affairs, user experience, workflow

## Abstract

**Background:**

Electronic health record (EHR) transitions are increasingly widespread and often highly disruptive. It is imperative we learn from past experiences to anticipate and mitigate such disruptions. Veterans Affairs (VA) is undergoing a large-scale transition from its homegrown EHR (CPRS/Vista) to a commercial EHR (Cerner), creating a unique opportunity of shedding light on large-scale EHR-to-EHR transition challenges.

**Objective:**

To explore one facet of the organizational impact of VA’s EHR transition: its implications for employees’ roles and responsibilities at the first VA site to implement Cerner Millennium EHR.

**Design:**

As part of a formative evaluation of frontline staff experiences with VA’s EHR transition, we conducted brief (~ 15 min) and full-length interviews (~ 60 min) with clinicians and staff at Mann-Grandstaff VA Medical Center in Spokane, WA, before, during, and after transition (July 2020-November 2021).

**Participants:**

We conducted 111 interviews with 26 Spokane clinicians and staff, recruited via snowball sampling.

**Approach:**

We conducted audio interviews using a semi-structured guide with grounded prompts. We coded interview transcripts using a priori and emergent codes, followed by qualitative content analysis.

**Key Results:**

Unlike VA’s previous EHR, Cerner imposes additional restrictions on access to its EHR functionality based upon “roles” assigned to users. Participants described a mismatch between established institutional duties and their EHR permissions, unanticipated changes in scope of duties brought upon by the transition, as well as impediments to communication and collaboration due to different role-based views.

**Conclusions:**

Health systems should anticipate substantive impacts on professional workflows when EHR role settings do not reflect prior workflows. Such changes may increase user error, dissatisfaction, and patient care disruptions. To mitigate employee dissatisfaction and safety risks, health systems should proactively plan for and communicate about expected modifications and monitor for unintended role-related consequences of EHR transitions, while vendors should ensure accurate role configuration and assignment.

**Supplementary Information:**

The online version contains supplementary material available at 10.1007/s11606-023-08282-5.

## Introduction

Electronic health record (EHR) transitions—a healthcare system’s efforts to replace its existing EHR system(s) with another—are becoming increasingly widespread.^1^ EHR transitions tend to be highly disruptive, affecting health systems’ productivity, efficiency, care quality, and employee satisfaction.^2–4^ One major organizational consequence of EHR transitions is their impact on employees’ ability to perform work duties in accordance with their roles and responsibilities. Clear, well-defined relationship between professional tasks and EHR roles is crucial to effective coordination between healthcare professionals.^5^ It is, therefore, imperative that systems undergoing EHR transitions learn from past lessons to effectively mitigate adverse effects.

Transitions from paper-based charts to EHRs in the 1990s–2000s were accompanied by significant changes to employee roles. Certain tasks were rendered obsolete, new tasks created, and existing roles and workflows (e.g., interprofessional division of labor) reconfigured.^6,7^ The transformative nature of EHR-to-EHR transitions demonstrated an effect on workflows, potentially leading to more/new work for clinicians^8^, cumbersome workflows^9^, negative user emotions^10^, and unexpected power structure changes.^11^ Given the breadth of potential unanticipated adverse consequences, it benefits health system leadership to learn from prior transitions so that they can proactively anticipate and address challenges related to roles and role changes. Learning from diverse health systems’ experiences enhances the field’s knowledge of EHR transitions comprehensively.

Veterans Affairs (VA), the USA’ largest nationally integrated health system, is in the early stages of a large-scale EHR transition from its homegrown EHR (CPRS/VistA, hereinafter “CPRS”) to the commercial Oracle-Cerner corporation’s Cerner Millennium. This is the largest EHR transition in history, already costing $1.8 billion with an overall projected cost of ~ $39 billion.^12^ The goal is to improve patient care quality and safety by better integrating documentation and operations between VA Medical Centers (VAMCs) and the Department of Defense. VA’s experience presents an unprecedented opportunity to expand the field’s understanding of large-scale EHR transitions and their implications for employee roles. We explore employees’ perspectives of how the EHR transition affected roles and responsibilities at the first VA EHR transition site as well as perceptions of the impact of role-related issues on communication, care safety, and care coordination.

## Methods

### Study Design

This work is part of a larger, ongoing multi-year evaluation of VA’s transition, EMPIRIC (*E*HR *M*odernization *P*artnership *I*ntegrating *R*apid Cycle Evaluation to *I*mprove *C*erner Implementation). EMPIRIC uses quantitative and qualitative methods to understand user EHR transition experiences. The evaluation was designated as quality improvement activity by the VA Bedford Healthcare System.

### Site

We collected data over 12 months around the new EHR implementation date (October 2020, or “go-live”) at Mann-Grandstaff VAMC in Spokane, WA (Spokane), the first VAMC to transition EHRs.

### Data Collection

After securing site leadership support, we contacted service leads to identify frontline clinicians and staff for interview recruitment. Snowball sampling identified additional participants.

We conducted a total of 111 interviews with participants at multiple windows: pre-go-live (July 2020-September 2020), during go-live (October 2020), post-go-live (December 2020), and ~ 12 months post-go-live (October 2021-November 2021) (Table 1). During go-live, we conducted brief (15–30 min) interviews to minimize participant burden during a high-stress period^13^; the rest were ~ 60 min.

**Table 1:** Interviews Conducted

	Pre-go-live interviews	Check-ins	2-month post-go-live interviews	1-year post-go-live interviews	Total
Leadership and clinicians*	11	22	14	13	**60**
Nurses**	6	12	4	5	**27**
Staff^†^	4	13	5	2	**24**
**Total (*n* = 26)**^**‡**^	**21**	**47**	**23**	**20**	**111**

^*^Physicians, clinical pharmacists, psychologists. Categories merged due to VA role fluidity and dual roles

^**^RNs, LPNs

^†^Medical assistants, phlebotomists, counselors, audiologists, physical therapists

^‡^Given site’s small employee body, broad participant categories preserve participant anonymity

Interview guides (Appendix 1) incorporated questions reflecting a priori and emergent domains of interest. Some domains (including roles) were identified in formative stages of the evaluation through team discussions on high-priority areas; others were added iteratively during inductive/deductive coding and analysis of 11 scoping interviews (not included in this paper’s dataset).

All interviews were conducted remotely via MS Teams™ by team members with experience in semi-structured interviewing. Prior to interviews, interviewees received information about the evaluation’s objective, methods, their rights as participants, and provided verbal consent. To increase participant comfort, we paired participants with the same interviewer whenever possible. During the interview, interviewers used grounded probes^14^ to elicit additional information and clarification. Interviewers completed post-interview debrief notes summarizing content and emerging reflections, which were subsequently discussed by the full team.

### Participants

We conducted 111 interviews with 26 frontline clinicians (*n* = 21) and staff (*n* = 5) (Table 1).

### Data Analysis

Interview transcripts were coded in ATLAS.ti 9 qualitative data analysis software using both a priori and emergent codes. A team of ten qualitative methodologists extracted coded passages relevant to work roles within EHR transitions. Qualitative content analysis^15^ was conducted to identify themes in participants’ accounts. Coding consisted of a focused analysis of interview passages related to roles. Responses to core interview questions and broader “time permitting” questions (e.g., “what else should we know that we have not asked about?”) were analyzed.

## Results

VA clinicians and staff perceived the setup of EHR roles as profoundly disruptive, affecting their ability to complete work, provide safe high-quality care, and feel satisfied with their work. We detail four key themes in employees’ accounts: (1) roles-light and roles-heavy systems; (2) erroneous work assignment; (3) changes to worker’s scope of practice; and (4) hindered communication between individuals with different roles. Illustrative quotes are provided (with interviewee ID, role, and time point).

### “Roles-Light” and “Roles-Heavy” Systems

In VA’s homegrown EHR, CPRS, all clinical data can generally be accessed by any user, with access to functions like medication and test ordering assigned by global training (e.g., licensed prescriber, nurse, clerk, pharmacist). User activity audits discourage inappropriate usage. Some role-based restrictions did exist in CPRS. However, CPRS can be described as largely “roles-light” compared to “roles-heavy” Cerner, where the entirety of EHR permissions and privileges is determined by highly specific user roles. A single site using Cerner could have 200 EHR user roles, each associated with a specific range of tasks and a role-specific interface. This contrast between CPRS and Cerner was noticed by participants. One interviewee shared:

“Everything’s so role-specific in Cerner. Not only what views you can see, but what your privileges are <…> everything was really built out with those roles. So it’s important to make sure that they’re assigned to the right experience” (*D103_NurseCaseManager. 10-month post-go-live*)*.*

### Erroneous Role Assignment

A major source of concern for clinicians and staff was inappropriate role assignment. After the transition, some clinicians were incorrectly assigned EHR roles that did not fit their actual clinical role. In some cases, clinicians lost the ability to complete necessary work tasks. In others, clinicians gained the ability to perform tasks inappropriate for their scope of practice. Such inaccurate role configuration had immediate negative implications for patient safety (e.g., some nurses lost the ability to order flu shots while also gaining the ability to submit narcotics orders):

“We were hoping with Cerner <…> if someone is trying to prescribe out of their scope <…> it would say, ‘you are unable to put in that order.’ But we’re actually able to put them in, with the provider’s signature, as long as they mark it as per clinical algorithm <… we> can’t do something simple like a flu shot, but we *can* prescribe Oxycodone” *(D79_NurseCaseManager. 10-month post-go-live)*.

Participants later clarified that the medication order permissions issue had been corrected, with incorrect role assignment identified as the cause.

“I think they found out that <order medication permissions> had to do with <individual role assignment. Even one of our newer nurses that just started, she came in and was put in as a physician, so she was also able to put in med orders <…> If we have anyone that’s new, we have to make sure that they’re actually assigned to the correct role.” *(D79_NurseCaseManager. 10-month post-go-live).*

In other cases, inappropriate role assignments persisted, creating confusion and dissatisfaction:

“It says that I’m a physician <…> So now some Nurse Practitioners are Nurse Practitioners, and some of us are still physicians. I think it’s not acceptable whatsoever. It says in there I’m a physician, and I’m not.” *(D78_NursePractitioner. 2-month post-go-live).*

A distinct, yet related variation of this issue concerned individuals with specialized, idiosyncratic VA roles. Exact equivalents of these roles—e.g., a specialty clinic ambulatory RN—did not exist in Cerner. Consequently, the individuals were granted access to a limited tasks which precluded their usual work.

“We’re in the Specialty Clinic, and we don’t fit into their box. <…> They think that <my> role is Ambulatory RN, so an Ambulatory RN needs these privileges. Well, no, I’m a Coordinator for a specialty service…” (*D61_RN. 2-week post-go-live).*

### Changes to Work Duties

Separate from incorrect EHR role assignments, some participants reported enduring changes to their responsibilities. In some cases, tasks previously within an individual’s scope of practice were taken away. Some responsibilities formerly held by RNs, such as running reports on narcotic prescriptions, shifted to clinicians, raising concerns about workloads from shifts in EHR-assigned responsibilities:

“We have our diabetic registries, opioid registries, that we were managing, but we don’t have access to those portals anymore <…> and there’s no way for us to get access to those things anymore. <…> We haven’t even seen what it looks like in 2 months, it’s kind of scary. And then with that, the nursing staff was doing the <prescription drug monitoring programs…> we were running those reports, but now in Cerner, that’s a provider only role <...> that was a big workload shift, it’s something that needs to be monitored annually, really strictly, it’s now another thing on the provider’s plate that the nursing staff was doing” *(D79_NurseCaseManager. 2-month post-go-live).*

A related concern was delays in care processes resulting from changed responsibilities.

“We have to communicate back and forth with this assistant all day long and say, check him in, check him out <…> and we can’t move forward until they’re checked in... this was not necessary before Cerner” *(D24_ClinicalPharmacist. 1-month post-go-live).*

“Some of our staff members aren’t able to put in orders <…> so they’re sending all of those simple orders to <providers>. The providers already have their workload, and now they have like an influx of 20 shingles vaccines, or 30 flu shot vaccines that they have to put orders in for” *(D79_NurseCaseManager. 2-month post-go-live).*

Some licensed practice nurse (LPN) tasks were also taken away or required new additional authorization:

“There’s a process for ordering durable medical equipment, a blood pressure cuff for instance. If I put in the particular type of order, and if my provider was to say, ‘you can order that blood pressure cuff,’ I don’t have the option of ‘ordering it per protocol, algorithm’, or whatever. It has to go to the provider to sign it off <…> Apparently RNs and providers have that option to sign it off and make it actionable, yet the LPN role does not” (*D100_LPN. 10-month post-go-live).*

Many LPNs found themselves unable to order lab tests, supplies or equipment which they previously could. Like RNs, LPNs worried about increased burden on their colleagues:

“<LPNs> used to be able to order stuff in CPRS. If we had somebody that came in for a catheter change and they were having symptoms of possible urinary tract infection, we could collect a urine at that appointment, put the order in the computer, and send it down to the lab, without having a doctor involved <…> But now, if that same scenario happened, we could collect the urine, put in the order, but then we have to wait for a provider to sign the order <…> So we’ve kind of lost the ability to just do simple things and it adds onto the providers tasks” (*D110_LPN. 10-month post-go-live).*

In other cases, enduring changes involved new tasks. Sometimes this was perceived merely as an inconvenience:

“There’s definitely things I have to do now because of Cerner, like review patient safety reports and complete the templates<...> and stuff like that, what weren’t part of my job before” (*D99_SiteLeader. 1-month pre-go-live).*

Other times, these new tasks were perceived as safety concerns, such as when new tasks fell outside of previous scopes for LPNs, a critical VA patient care support role. For example, to address rising concerns about data integrity, LPNs were assigned two new types of intake tasks, medication reconciliation and mental health screenings, which they were not always prepared to perform.

“Some of the screening questions that the LPNs were performing, they were pretty uncomfortable with. <Like> the mental health questions, while they’re just doing their intake <…> If <a patient is> positive on any of the questions, that sends up a red flag, <but> it doesn’t alert the provider. So, when the providers start seeing the patients, they don’t see these positive responses <...and LPNs> felt like maybe it was out of their scope*” (D40_MSA. 1-month pre-go-live).*

This new arrangement increased the workloads for both LPNs (not trained in this task) and providers (who needed to proactively check for positive screens due to the lack of alerting function), as well as heightened the risk of missed positive screens.

Overall, LPNs felt their role and professional scope was particularly affected by, and not understood through, the system change. Historically, VA LPNs have had broader (yet legal) duties than private practice counterparts. Lack of an appropriate LPN assignment in Cerner led LPNs to feel undervalued, unable to carry out usual tasks, and concerned about the potential harm to patients:

“I was on a committee for a short period of time on <how the LPNs will fit>, what we can and can’t do. And we still don’t know all of that yet. But when I went to my training <the trainer> made the comment, “this is for the RNs”. <You’ve> got to include the LPNs, we’re not an afterthought there, we are a big part of this <…> So that’s kind of frustrating, that we don’t know how this is going to work for us, what we can and can’t do as far as charting and how we’re going to get our job done. So if we don’t have access to <all of that> how do we do our jobs?” *(D5_LPN. 1-month pre-go-live).*

### Communication Hindered Between Individuals with Different Roles

The EHR transition changed work communication and coordination between individuals with different EHR roles. Whereas CPRS’s interface looks nearly identical for all users, Cerner’s interface varies significantly depending on role. Users reported that these differences hindered their ability to receive formal *and* informal support for learning the new EHR.

“[Everyone] who’s a nurse, versus a doc, versus a pharmacist gets a slightly different view of PowerChart, so that’s <...> what’s being taught. So really, somebody will get one thing out of a training, and another person will get another thing out of a training, and it may or may not come back to the services and be exchanged” *(D95_ClinicalPharmacist. 10-month post-go-live).*

Once Cerner went live, participants reported that charts looked different based on department, and that workflows for the same task could vary significantly depending on role. Users in different roles could not walk each other through creating outpatient requests or orders, limiting informal peer-to-peer support.

**“**Because we all have different ‘roles,’ we also see how Cerner actually looks to each of us is different. So if I log in, versus a Urologist, versus an Orthopedic Surgeon, there’s slight variations in the tabs <…> which is very odd to me <because> we’re all just doctors <...> So that causes more problems with communicating about how to do something” (*D107_Surgeon. 10-month post-go-live*).

Moreover, interfaces differences were described as an impediment to broader facility-wide communication. Participants reported less virtual collaboration and communication. Individuals were siloed, even within small teams, due to the segmentation of duties and more narrowly defined roles.

“We’re collaborating less, <…> we’re talking about clients, veterans, less holistically and working together as a team because of the way this chart is set up. Now it <makes it seem> as if all of the service lines are really separate and there’s no need for us to collaborate about what treatment the veteran is getting” *(D88_Therapist. 2-month post-go-live)*.

## Discussion

In our evaluation of a large-scale EHR transition in the VA, we explored how adopting a new EHR may result in unexpected and far-reaching changes in employees’ work, disrupting role clarity and scope, division of labor, and established communication patterns (Table 2). These findings carry implications for research on EHR transitions and contain lessons for the increasing number of healthcare systems undergoing such transitions and EHR vendors partnering with them. As of April 2023, VA’s EHR transition is in a state of “reset” due to lingering concerns about the new EHR system’s safety and usability. During this time, VA is well-positioned to learn from the experiences of the sites that have transitioned to Cerner, including Spokane, to proactively anticipate and ameliorate negative effects of role-related issues on clinical care and safety, clinician communication, and task completion.^10,11,16,17^ VA’s experiences also contribute to the case diversity of the growing field of research on EHR transitions and can be drawn on by other health systems.

**Table 2:** Key Themes and Examples

Key theme	Example	Further reading	Potential mitigation strategy
“Roles-light” and “roles-heavy” systems	Variation in granularity of role-assigned privileges	(Blobel, 2004)^33^	Prepare users for degree of change
Erroneous work assignment	Nurse practitioner assigned physician role	(Sittig, Belmont & Singh, 2018)^34^	Refine mechanisms for identifying inappropriate assignments (e.g., ticketing)
Changes to worker scope of practice	User newly responsible for reviewing patient safety reports	(Carayon et al.,, 2015)^17^	Maintain inventory of official/unofficial user tasks
Hindered communication between individuals with different roles	Variations in users’ interfaces	(Adler-Milstein & Wang, 2020)^35^	Provide additional training for users in peer support positions

The first prominent concern was the disruptive nature of transition from a “roles light” system to a “roles-heavy” one. EHR transitions are already disruptive; transitions between EHRs structured in foundationally different ways add an additional layer of complexity. Users should be given realistic communication about forthcoming transitions, particularly when switching to an EHR with a substantially different assignment approach.

The second concern was erroneous role assignment. Role-based EHR access seeks to ensure patient data safety and appropriate patient care. Incorrect role assignment creates immediate threats to both.^18,19^ Users without prescribing privileges being able to submit medication orders, especially controlled substances, is particularly concerning, though we note such situations were corrected. Such issues, if not promptly identified and resolved, may threaten the quality and safety of patient care.^20^ Furthermore, the emotional effects of disrupted work can impact employee confidence and morale, leading to burnout and turnover.^21^

It is essential that future VA sites—as well as non-VA systems planning EHR transitions—mitigate the challenge of incorrect role configuration. Both healthcare systems and EHR vendors have contributions to make. Healthcare systems may refine mechanisms for identifying inappropriate assignments. In Spokane, the ticket process was a primary mechanism to address improperly configured roles and experienced improvement efforts (e.g., building a dashboard to improve transparency on configuration resolution status; prioritizing role configuration tickets that affected quality, safety, and efficiency of care; re-engineering change request processes).^22^ Other health systems may consider similar approaches. Meanwhile, EHR vendors should commit to mitigating human error in assigning roles while instituting processes for promptly correcting inappropriate assignments.^23^

The issue of enduring changes to employees’ scope of duties in the aftermath of the EHR transition is distinct from the previously discussed problem of incorrectly configured EHR roles. The first case issue stems from incorrect assignment of EHR roles to specific users and/or erroneous expansion/narrowing of roles—unintended problems subsequently corrected. In the latter case, however, the EHR role assignment *codified* seemingly lasting transformations to the employees’ task scope and division of labor. This phenomenon is best understood in VA’s larger historical and organizational context. Since 2016, VA has sought standardization in clinical duties and privileges. This initiative to implement national standards of practice has targeted over 50 clinical occupations.^24,25^ For some occupations, standards have been approved and published in the Code of Federal Regulations^26^; for others, the work is ongoing^27^. The push for greater uniformity in standards of practice within VA was accelerated by the EHR transition. It appears, however, that the intention to leverage the EHR transition to support the ongoing standardization of clinical roles was not effectively communicated to some targeted work groups. Most prominently, LPNs seem to have experienced profound and unexpected transformations in their task scope.

Some negative consequences of leveraging the EHR transition to promote standardization of roles may be prevented or ameliorated through a detailed inventory of individuals’ official and tacit tasks and responsibilities to ensure accurate reflection in EHR permission and privilege setup. This includes considering the needs of employees with dual roles (e.g., clinician-administrator), as well as explicitly recognizing key roles within facilities not included within the new EHR. Where changes to the employees’ duties are unavoidable due to the particularities of EHR setup and/or the institution’s desire to make changes to the roles themselves, healthcare systems must facilitate team-specific retraining long before the new EHR is introduced in patient care, benefitting both employees and patients. Overall, the effects of enduring changes in roles due to the EHR remain to be seen; paper-to-EHR transitions suggest that while benefits may ultimately result from transitions (e.g., improved work flow^28^, improved documentation^29^), these may only emerge in the long term.

Finally, we found that participants perceived the new EHR as affecting communication between individuals, even in the same patient encounter. In VA’s new EHR, role interfaces highlight some patient information and/or workflows while obscuring others (e.g., behind roundabout interface navigation or nestled within tabs). This affects users’ distribution and content of work tasks and information flows^16,30,31^ and has further implications for systems at large, creating silos and disparities in information access and work processes.^32^

Differences in screen appearance and divergent EHR-based workflows are deterrents to successful peer-based support, a well-documented strategy for EHR-based learning.^33^ When configuring roles, effective communication between staff with different roles should be supported.^35^ Participants also found that users with additional training (including an understanding of differences in role-based views) often reduced the need for formal channels of issue resolution (e.g., tickets, change orders).

Our findings raise questions about the degree of fit between the role-based approach and the needs of a complex healthcare system like VA. VA sites and clinics have significant leeway in deciding how best to organize and distribute work among healthcare professionals. VA’s homegrown EHR has facilitated this arrangement due to its predominantly audit-based approach to EHR access and loose definition of roles. Conversely, Cerner presents a more regimented and standardized approach to EHR roles that may not account for unique VA clinician organizational needs. Role-based access is increasingly advocated as the preferred approach to ensuring EHR system security. A question our work raises, but does not resolve, is whether and *how* an EHR with a role-based access approach can be customized to fit the needs of a complex healthcare system like VA.

Our work has limitations. Data comes from VA employees who volunteered to participate amidst a challenging transition early during the COVID-19 pandemic; self-selection bias and compounding effects may have affected our data. This work was completed at the first VAMC to undergo EHR transition. Staff experiences at other VA sites and non-VA systems may differ.

## Conclusions

Health systems preparing to transition to a new EHR must anticipate substantive intended and unintended changes in professional roles. Solutions should be designed to fit the specific needs of employees, teams, and systems at large. Vendor delineated role assignments and configurations affect users profoundly. Our findings support the understanding that EHR transitions are deeply disruptive. A new EHR’s characteristics (e.g., increased role-based access, shifts in access, and interface layout) can result in unexpected changes to how users understand their roles, which tasks are done by whom, and how employees communicate with and help one another. To mitigate employee job dissatisfaction and patient safety risks that can accompany EHR transitions, healthcare systems and vendors should collaborate to ensure proper role assignment and resolve inappropriate role assignments.

### Electronic supplementary material

Below is the link to the electronic supplementary material.

Supplementary file1 (PDF 520 kb)

## Data Availability

Data analyzed in this study are not publically available due to potentially identifying and sensitive content from participants. Minimal de-identified aggregate data in the form of tables are available from the corresponding author on reasonable request and are subject to corresponding author's institutional approval.
